# Improving Inpatient Surveys: Web-Based Computer Adaptive Testing Accessed via Mobile Phone QR Codes

**DOI:** 10.2196/medinform.4313

**Published:** 2016-03-02

**Authors:** Tsair-Wei Chien, Weir-Sen Lin

**Affiliations:** ^1^ Chi Mei Medical Center, Taiwan Research Department Chi Mei Medical Center, Taiwan Tainan Taiwan; ^2^ Chia-Nan University of Pharmacy and Science Department of Hospital and Health Care Administration Chia-Nan University of Pharmacy and Science Tainan Taiwan

**Keywords:** computer adaptive testing, patients’ experience, partial credit model, consultation experience and perception, smart phone

## Abstract

**Background:**

The National Health Service (NHS) 70-item inpatient questionnaire surveys inpatients on their perceptions of their hospitalization experience. However, it imposes more burden on the patient than other similar surveys. The literature shows that computerized adaptive testing (CAT) based on item response theory can help shorten the item length of a questionnaire without compromising its precision.

**Objective:**

Our aim was to investigate whether CAT can be (1) efficient with item reduction and (2) used with quick response (QR) codes scanned by mobile phones.

**Methods:**

After downloading the 2008 inpatient survey data from the Picker Institute Europe website and analyzing the difficulties of this 70-item questionnaire, we used an author-made Excel program using the Rasch partial credit model to simulate 1000 patients’ true scores followed by a standard normal distribution. The CAT was compared to two other scenarios of answering all items (AAI) and the randomized selection method (RSM), as we investigated item length (efficiency) and measurement accuracy. The author-made Web-based CAT program for gathering patient feedback was effectively accessed from mobile phones by scanning the QR code.

**Results:**

We found that the CAT can be more efficient for patients answering questions (ie, fewer items to respond to) than either AAI or RSM without compromising its measurement accuracy. A Web-based CAT inpatient survey accessed by scanning a QR code on a mobile phone was viable for gathering inpatient satisfaction responses.

**Conclusions:**

With advances in technology, patients can now be offered alternatives for providing feedback about hospitalization satisfaction. This Web-based CAT is a possible option in health care settings for reducing the number of survey items, as well as offering an innovative QR code access.

## Introduction

Patient reports are central to the evaluation of medical care, both in terms of treatment outcomes (ie, patient-reported outcomes and in terms of experiences of quality of care (ie, patient-reported experience measures) [1]. A quality standard for patient experience in the United Kingdom’s National Health Service (NHS) has been developed by the National Institute for Health and Care Excellence [2,3]. The UK National Adult Inpatient Survey—UK NHS 70-item questionnaire—has been in use in Great Britain since 2002, gathering data from over 620,000 patients every year [4].

The first public reporting of the US equivalent of such surveys, the Hospital Consumer Assessment of Healthcare Provider and Systems (HCAHPS), began in 2008. HCAHPS collects more than 3.0 million completed surveys from 3912 hospitals every year. On average, more than 28,000 patients are surveyed every day about their recent hospital experience, and more than 8400 patients (approximately 30% response rate) complete the HCAHPS inpatient survey every day [5].

### Research Questions

A concern reported in the literature is the burden on patients of answering all survey questions at one time [6-9]. More than 3.6 million patients completed mail-in surveys from the UK NHS (600,000 patients). The US survey (3.0 million respondents) required approximately 6 hours of time per month and cost approximately US $70 per month [3] to examine people’s health service experiences [10]. However, the UK NHS 70-item questionnaire is significantly longer than the US HCAHPS 25-item survey [11,12]. To reduce patient burden, it is first necessary to shorten the item length of the UK NHS inpatient questionnaire to increase response rates without compromising its assessment reliability [2,13].

Many studies [6-9] have reported that item response theory (IRT)-based computer adaptive testing (CAT) has the advantages of both long-form and short-form questionnaires [14-16] in precision and efficiency. Since many patients (or their guardians) already own mobile phones, which they are comfortable using, it makes sense to use them in hospitals and for hospital surveys. At this time, no studies have been published reporting online CAT via mobile phones in medical fields.

However, many skip items (see Multimedia Appendices 1 and 2) exist in the UK NHS 70-item questionnaire, which can be confusing and may perplex researchers on CAT implementation. Thus, our second aim was to tackle the problem of skip items in the UK NHS questionnaire and to implement the online CAT.

### Rasch Partial Credit Model Applied to the Item Response Theory–Based Computer Adaptive Testing

Many researchers have contributed to the dichotomous [6] and polytomous [7-9] formats used by CAT. The UK NHS questionnaire comprises items with different categories (eg, 3 and 6 categories for Items 40 and 41; see Multimedia Appendix 1). It is suited for applying the Rasch partial credit model (PCM), that is, items with a different number of responses and with an equal discrimination parameter [17], or the generalized partial credit model, that is, items with a different number of responses and with unequal discrimination parameters [18], if those items form a unidimensional construct. None was jointly available for a comparison of precision and efficiency differences of CAT estimation with the aforementioned methods commonly used in literature, such as PCM, answering all items (AAI), and the randomized selection method (RSM).

Further, as mobile phones have become ubiquitous in the health care setting [19], it is important to offer an alternative online Rasch PCM-CAT assessment to gather hospitalization experience feedback from patients. We propose access to the questionnaire using a quick response (QR) code via mobile phone.

### Aims of this Study

The aims of the current study were to investigate whether CAT can (1) be efficient with item reduction and (2) be used with QR codes used for mobile phones.

## Methods

### Study Data

The UK NHS 70-item questionnaire regarding patient experience was downloaded from the NHS official website [11]. The item and its threshold difficulties (lower summation scores for an item imply that it was more difficult for examinees to respond) were roughly determined by hand computation according to the key findings report for the 2008 inpatient survey [20]. We simulated an interactive metric of 1000 persons (following a normal distribution [~*N*(0,1)], called true scores) and 70 items (estimated with aforementioned item difficulties) using the Rasch PCM model [17,21]. Nine items originally designed to automatically select different paths were set with different probabilities by the authors. The remaining 61 items were allocated different weighted scores (see Multimedia Appendix 1). A set of 24 items (ie, regarding sections of the ward, doctors, nurses, patient care, and treatment) was extracted from the UK NHS 70-item questionnaire to be the CAT item pool (see Multimedia Appendix 2). We assumed that the set of 24 items is unidimensional based on the report from the previous study paper [13]. Because these 9 conditional selection path items make CAT difficult to design for a computer, they were excluded from the CAT item pool. Multimedia Appendix 3 shows the file layout and fields we designed for use with the datasets.

### Unidimensionality

The Rasch model, named after Georg Rasch [22], is a psychometric model for analyzing categorical data as a mathematical function of the trade-off interaction between (1) the respondent’s latent trait (eg, hospitalization perception level in this study) and (2) the item difficulties. The dichotomous Rasch model and its extensions (eg, family models: rating scale model [23], PCM [17]) are successfully used in other areas, including the health profession [24] and market research [25], because of their general applicability [26].

The study data need to meet the following criteria to fit the Rasch model: the infit and outfit mean square errors (MNSQ) of all items are ˂1.5 for unidimensionality and ˃0.5 for local independence [27]. Simulation data were generated fitting to the Rasch PCM model [21].

### Task 1: Investigating Computer Adaptive Testing Efficiency and Accuracy

Three scenarios were designed to compare their efficiency and accuracy on the UK NHS 70-item questionnaire: (1) the AAI (answering all items on those 24 items), (2) the RSM (randomized selection method to draw 12 items), and (3) the CAT (at least 5 items and stop at person reliability of 0.80) responding to the 24-item pool.

We applied CAT stop rules, such as when person reliability reaches 0.80 (=[1 − SEM_pi_]×[1 − SEM_pi_], where SEM_pi_=person standard error of measurement on item i=1/variance_pi_=1/information_pi_), and when the last 5 average consecutive person estimation change is less than 0.05 after the minimum necessary completed number of items is ≥5.

**Figure 1:**
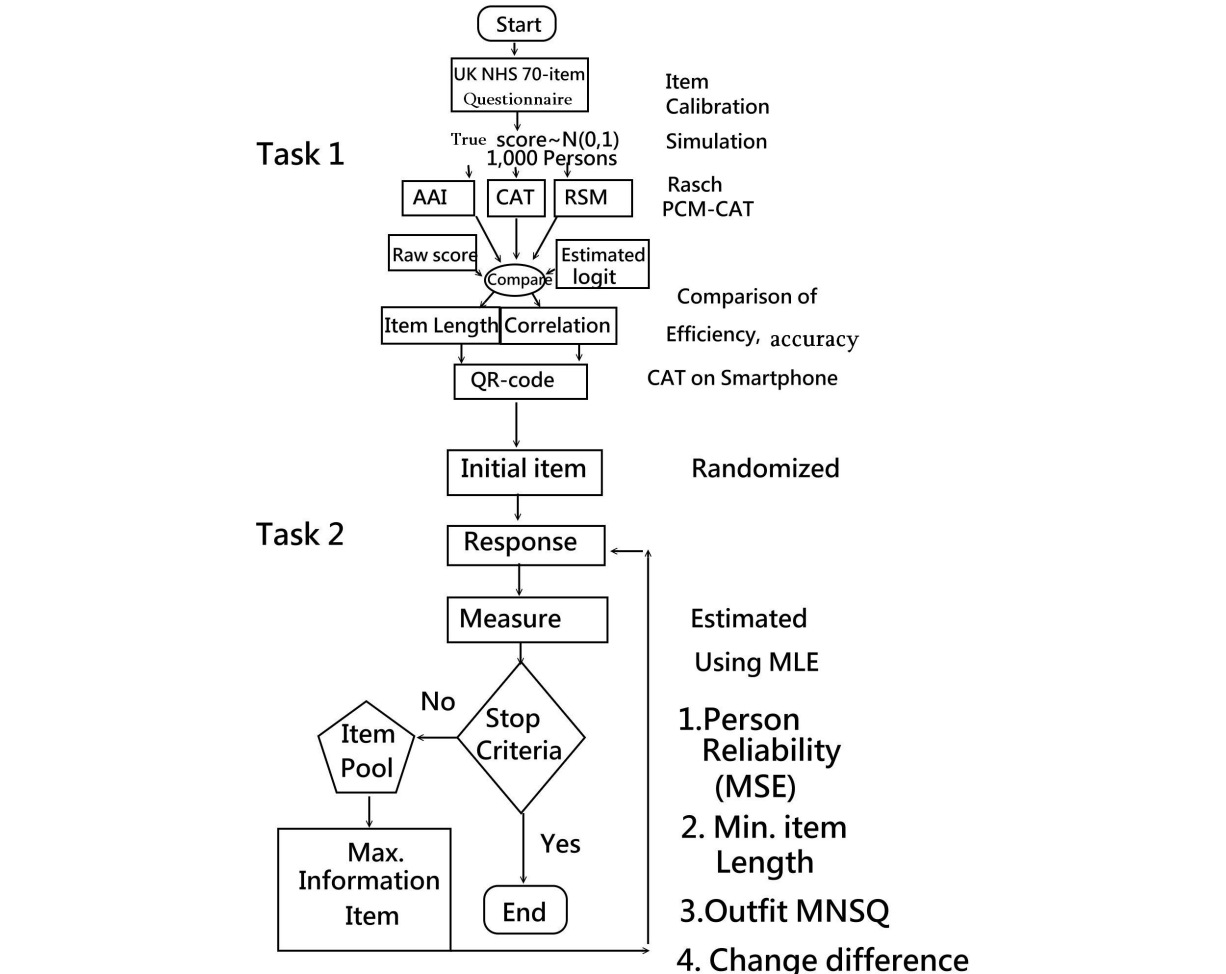
Study flowchart.

In addition, we ran an author-made VBA (Visual Basic for Applications) module in Microsoft Excel to conduct a simulation study (see Multimedia Appendix 4). Another Web-based CAT was programmed for use on mobile phones. The maximum likelihood estimation algorithm [28] (see Multimedia Appendix 4) was used to (1) estimate person measures on the three scenarios, (2) compute correlation coefficients between estimated person measures among the three scenarios and the original true scores to verify CAT accuracy, and (3) analyze CAT efficiency of item length shortened by CAT compared with the other two scenarios (ie, AAI and RSM) (see Figure 1).

### Task 2: An Online Assessment Using Mobile Phones

#### The Conditional Path Skip Items Designed on UK NHS-70

The path skip item was automatically redirected to the next according to the respective route designed in the field of the item dataset (see Multimedia Appendix 3). To illustrate Item 39 in Multimedia Appendix 1, two route fields were filled with Items 40 and 41 in response to the respective answer code (eg, 1 or 2). In contrast, the route fields for those ordinary non-skip items were kept empty (or a null value).

#### An Online Computer Adaptive Testing Routine for Gathering Feedback From Patients

An online routine was designed for patients to report their perceptions of their inpatient hospitalization experience. The UK NHS 70-item questionnaire (see Multimedia Appendix 2) was uploaded to website. The first CAT item is randomly selected from the item pool (ie, Items 15-38) after Item 14 is answered. The next item to be answered is the item with the maximal variance in the remaining items according to the provisional person ability [7,29]. Multimedia Appendix 5 shows details on the item selection rules and the Excel VBA codes for the conditional path items. All the responses are then automatically saved on the study website (see Multimedia Appendix 3).

## Results

### Items Fit to the Rasch Model

The set of 24 items (see Table 1) was taken as unidimensional due to simulation data fitting the Rasch model’s requirement with values of infit and outfit MNSQ between 0.5 and 1.5 [21]. Each item has its own threshold difficulties (see Multimedia Appendix 4).

**Table 1:** The 24 items selected from the UK NHS 70-item questionnaire.

No.	Item	Threshold^a^	Difficulty^b^	In MNSQ	Out MNSQ
15	Were you ever bothered by noise at night from other patients?	1	2.17	0.96	1.00
16	Were you ever bothered by noise at night from hospital staff?	1	1.84	1.03	1.11
17	In your opinion, how clean was the hospital room or ward that you were in?	3	-0.56	0.97	0.98
18	How clean were the toilets and bathrooms that you used in the hospital?	3	-2.20	0.97	0.98
19	Did you feel threatened during your stay in the hospital by other patients or visitors?	1	1.88	0.97	0.98
20	Were hand-wash gels available for patients and visitors to use?	2	1.33	1.02	1.02
21	How would you rate the hospital food?	3	-1.72	0.96	1.02
22	Were you offered a choice of food?	2	-0.04	0.98	0.98
23	Did you get enough help from staff to eat your meals?	2	0.98	1.02	1.01
24	When you had important questions to ask a doctor, did you get answers that you could understand?	2	-1.01	1.03	1.01
25	Did you have confidence and trust in the doctors treating you?	2	-0.86	1.06	1.07
26	Did doctors talk in front of you as if you weren’t there?	2	-1.15	0.96	1.00
27	When you had important questions to ask a nurse, did you get answers that you could understand?	2	1.48	0.99	0.99
28	Did you have confidence and trust in the nurses treating you?	2	1.26	1.01	1.01
29	Did nurses talk in front of you as if you weren’t there?	2	-1.46	1.04	1.05
30	In your opinion, were there enough nurses on duty to care for you in the hospital?	2	-1.40	0.94	0.92
31	Sometimes in a hospital, a member of staff will say one thing and another will say something quite different. Did this happen to you?	2	-1.22	0.96	0.95
32	Were you involved as much as you wanted to be in decisions about your care and treatment?	2	0.56	1.02	1.02
33	Did you have confidence in the decisions made about your condition or treatment?	2	-0.78	0.96	0.96
34	How much information about your condition or treatment was given to you?	2	1.48	1.04	1.04
35	Did you find someone on the hospital staff to talk to about your worries and fears?	2	-0.77	1.04	1.04
36	Do you feel you got enough emotional support from hospital staff during your stay?	2	-0.75	0.96	0.95
37	Were you given enough privacy when discussing your condition or treatment?	2	1.58	0.98	0.98
38	Were you given enough privacy when being examined or treated?	2	-0.65	1.01	1.02

^a^Threshold denotes the number of categories on each item, for example, 2 for three categories and 1 for two categories.

^b^Difficulty represents item difficulty in a unit of logit (=log odds).

### Task 1: Investigating Computer Adaptive Testing Efficiency and Accuracy

Table 2 indicates that the CAT relates the true scores (*r*=.97 in column 2) and the AAI (*r*=.97 in columns 3 and 4) to a high association, indicating that the CAT earns an equivalent accuracy compared to the AAI and a higher accuracy than the RSM (in column 5 of the estimation section). The summation scores have a higher correlation (*r*=.98) to the within counterparts (eg, summation RSM scores vs estimated RSM logit scores) and a slightly lower correlation (*r*=.92-.97) to the between counterparts (eg, summation RSM scores vs estimated AAI or CAT logit scores), implying that the raw summation scores have a high correlation (*r*=.98) with the estimated logit scores shown in the last 4 columns of Table 2. The bottom row of Table 2 shows that the CAT earns the shortest item length, indicating the CAT has advantages in efficiency over AAI and RSM.

**Table 2:** Comparisons of efficiency and accuracy among the AAI, RSM, and CAT.

		Estimated logit scores				Summation scores	
		True score	CAT	AAI	RSM	RSM	AAI
True score			0.97	0.96	0.91	0.92	0.95
**Estimation**							
	CAT	0.97		[0.98]	0.93	0.94	0.97
	AAI	0.96	[0.98]		0.95	0.95	0.98
	RSM	0.91	0.93	0.95		[0.98]	0.94
**Summation**							
	RSM	0.92	0.94	0.95	[0.98]		0.96
	AAI	0.95	0.97	[0.98]	0.94	0.96	
	Mean	0.03	0.01	-0.06	-0.05	4.64	4.59
	Standard deviation	1.02	1.07	0.93	0.74	1.19	1.44
Item length^a^			40.50	57.31	45.42	45.42	57.31

^a^Item length denotes those items, excluding the 9 automatic-selection-to-different-path items.

### Task 2: Online Computer Adaptive Testing Assessment

**Figure 2:**
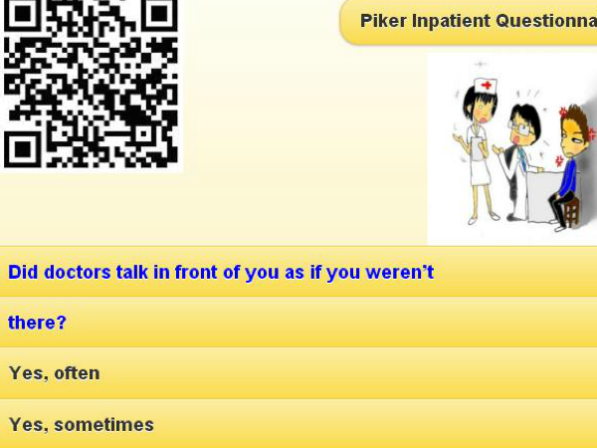
A snapshot of a QR code and the CAT item.

**Figure 3:**
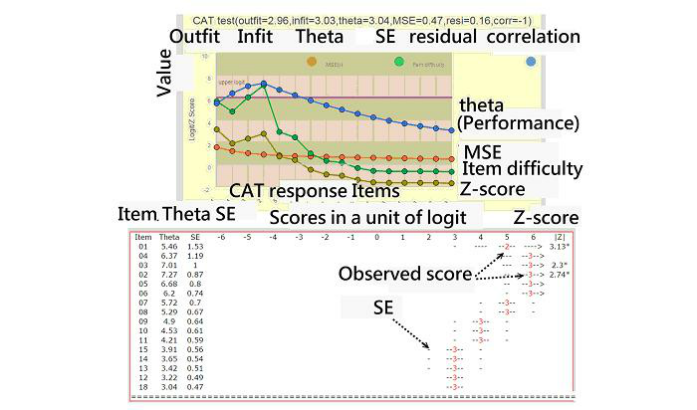
The process of CAT estimated scores.

By scanning the QR code (see Figure 2), the CAT icon appears on the patient’s mobile phone. The mobile CAT survey procedure was demonstrated item-by-item in action (see Figure 3). Person fit (ie, infit and outfit MNSQ) statistics showed the respondent behaviors. Person theta is the provisional ability estimated by the CAT module.

The standard error in Figure 3 was generated by the following formula (see Multimedia Appendix 5): 1/√(Σ variance(*i*)), where *i* refers to the CAT finished items responded to by a person [30]. In addition, the residual (resi) in Figure 3 was the average of the last five change differences between the pre-and post-estimated abilities on each CAT step. CAT will stop if the residual value is less than 0.05. “Corr” refers to the correlation coefficient between the CAT estimated measures and its step series numbers using the last five estimated theta (=person measure) values. The flatter the theta trend, the higher the probability that the person measure is convergent with a final estimation.

## Discussion

### Principal Findings

We verified that computer adaptive testing can be (1) efficient with questionnaire item reduction and (2) used with QR codes on mobile phones.

The CAT item pool was designed using Items 15-38 of the UK NHS 70-item questionnaire. We found that CAT can be more efficient for answering questions than both AAI and RSM without compromising its measurement accuracy, which is consistent with previous studies [6-9]. Our online CAT inpatient survey for gathering satisfaction responses from patients was accessed by scanning a QR code on a mobile phone, which has never been demonstrated previously.

Many studies have discussed patient perceptions about hospitals and the benefit of listening to other patient experiences when choosing a hospital [31,32]. There has been a rapid increase in the number of websites that allow patients to rate their hospitals [33,34]. Almost all health care providers have been explicitly required to conduct surveys of their patients’ health care experiences. However, those surveys often use an individual item-by-item approach to disclose patient views on hospital service quality, which does not provide hospital staff with information to make further improvements without considering the overall hospital performance [13].

### Implications and Future Considerations

We demonstrated that an NHS inpatient experience questionnaire with shortened items can be used with an IRT-based CAT technique without compromising its measurement accuracy. Using a CAT approach with such complex question structure jointly with item pools and conditional path skip items is rare. Our online CAT module used by scanning a QR code on a mobile phone can be extended to many dimensions simultaneously in a survey. For example, the Clinical Dementia Rating scale [35] used in patients with dementia consists of six domains. We could design a module using CAT through several procedures in a common questionnaire in the future.

### Strengths

Hospital staff must consider both the efficiency and utility of assessment for the selection of the CAT items [36]. The traditional survey collects all feedback from patients through particular sets of questions to assess what causes patient difficulty or dissatisfaction. The assessment results help hospital managers determine where improvements can be made [36]. We can use the Rasch simulation technique to overcome the problem in questionnaires of unanswered items (ie, which do not provide hospital staff with information to make further improvement). This Rasch simulation technique [21] can be used to fill in the expected responses to those unanswered CAT items according to the final person theta (ability) and the specified item difficulties. Thus, the CAT can provide efficient assessments and the full information needed to make improvements.

Furthermore, the person outfit mean square in CAT is also saved in our database (see Multimedia Appendix 3). An outfit mean square of 2.0 or greater for a patient indicates a possibly aberrant response pattern [37], such as cheating, careless responding, lucky guessing, creative responding, or random responding [38], which makes it hard to reveal valuable information using the traditional survey method.

### Limitations

Six limitations of this study are addressed. First, the study was based on the assumption of unidimensionality across those 24 CAT items. Although several articles have supported the notion that the UK NHS 70-item questionnaire can construct a one-dimension domain [13,31], those items cannot be generalized to the 24 CAT items used in different countries or by different groups. Future studies should further verify those 24 items to make the CAT module valid and feasible in health care practice.

Second, the first CAT item was selected from a randomized item pool. The CAT selection rule for the first item can be redesigned referring to the previously completed items and inferring a provisional theta (ie, person measure) to select the first item with the maximum information (ie, variance) in the item pool so that the questionnaire length could be shorter (see Multimedia Appendix 6).

Third, only one CAT module was designed in the NHS inpatient questionnaire due to the conditional selection path skip items that existed in non-CAT items. Future studies are recommended to overcome this barrier and to design a CAT-by-CAT approach in the questionnaire so as to reduce more item length in a questionnaire.

Fourth, we have not discussed the issue of participation options using traditional postal mail or email. Because not all patients possess a mobile phone, specifically a smartphone, and 3G/4G WiFi communication, all options (mail or email) must be offered to patients when invited to participate in the survey. Readers have found the email option useful to answer questions either by connecting to the Web, or by scanning a QR code on a mobile phone (eg, Figure 2) if applying the CAT demonstrated in this study. Future studies are needed to further explore and improve the processes of the CAT survey.

Fifth, we conducted a simulation of 1000 patients’ true scores followed by a standard normal distribution. This might contradict the general experience of satisfaction surveys with ceiling effects that impede standard normal distribution. Future studies are needed to sample from a negatively skewed population to further verify whether the CAT can be efficient on item reduction over AAI and RSM.

Sixth, the unidimensionality of the 24 CAT items may be questioned given the different realms of hospital ward, doctors, nurses, patient care, and treatment. It might be implausible to assume that these 24 items are unidimensional. Future studies are required to further investigate the issue.

### Conclusion

With advances in technology, we can now offer patients alternative ways via mobile phones to gather their feedback on hospitalization satisfaction. The online CAT can reduce the number of survey items for patients to respond to, as well as be accessed via mobile phone using a QR code.

## Appendix

Multimedia Appendix 1 A format of skip items in a questionnaire.

Multimedia Appendix 2 The UK NHS 70-item questionnaire.

Multimedia Appendix 3 The study item dataset.

Multimedia Appendix 4 Excel VBA module used for CAT simulation.

Multimedia Appendix 5 Excel module for simulation on CAT, AAI, and RSM saving to spreadsheet NAT.

Multimedia Appendix 6 Introduction to CAT.

## Abbreviations

AAI: answering all items
CAT: computer adaptive testing
HCAHPS: Hospital Consumer Assessment of Healthcare Providers and Services
IRT: item response theory
MNSQ: mean square
NHS: National Health Service
PCM: partial credit model
QR: quick response
RSM: randomized selection method
SE: standard error
SEM: standard error measurement
VBA: Visual Basic for Applications
