# Supplementary material for: Improving Inpatient Surveys: Web-Based Computer Adaptive Testing Accessed via Mobile Phone QR Codes
Source: JMIR Med Inform. 2016 Mar 2;4(1):e8. doi: 10.2196/medinform.4313 (PMC4795329; doi:10.2196/medinform.4313)
Supplement: Multimedia Appendix 1 [file medinform_v4i1e8_app1.pdf]

Multimedia Appendix 1. Skip items.

39. Were you ever in any pain?

1. ☐ Yes → Go to 40
2. ☐ No → Go to 41

40 Do you think the hospital staff did everything they could to help control your pain?

1. ☐ Yes, definitely (score 10 points)
2. ☐ Yes, to some extent (score 5 points)
3. ☐ No (score 0 point)

41. How many minutes after you used the call button did it usually take before you got the help you needed?

1. ☐ 0 minutes / right away (10 points)
2. ☐ 1-2 minutes (7.5 points)
3. ☐ 3-5 minutes (5 points)
4. ☐ More than 5 minutes (2.5 points)
5. ☐ I never got help when I used the call button (0 points)
6. ☐ I never used the call button
