# Supplementary material for: Improving Inpatient Surveys: Web-Based Computer Adaptive Testing Accessed via Mobile Phone QR Codes
Source: JMIR Med Inform. 2016 Mar 2;4(1):e8. doi: 10.2196/medinform.4313 (PMC4795329; doi:10.2196/medinform.4313)
Supplement: Multimedia Appendix 2 [file medinform_v4i1e8_app2.pdf]

INPATIENT QUESTIONNAIRE

What is the survey about?

This survey is about your **most recent** experience as an **inpatient** at the National Health Service hospital named in the letter enclosed with this questionnaire.

Who should complete the questionnaire?

The questions should be answered by the person named on the front of the envelope. If that person needs help to complete the questionnaire, the answers should be given from his / her point of view – not the point of view of the person who is helping.

Completing the questionnaire

For each question please cross ☒ clearly inside one box using a black or blue pen. For some questions you will be instructed that you may cross more than one box.

Sometimes you will find the box you have crossed has an instruction to go to another question. By following the instructions carefully you will miss out questions that do not apply to you.

Don’t worry if you make a mistake; simply fill in the box ■ and put a cross ☒ in the correct box.

Please **do not** write your name or address anywhere on the questionnaire.

Questions or help?

If you have any queries about the questionnaire, please call the helpline number given in the letter enclosed with this questionnaire.

Taking part in this survey is voluntary. **Your answers will be treated in confidence.**

Please remember, this questionnaire is about your **most recent** stay at the hospital named in the accompanying letter.

ADMISSION TO HOSPITAL

1. Was your most recent hospital stay planned in advance or an emergency?

1 ☐ Emergency or urgent ➔ **Go to 2**

2 ☐ Waiting list or planned in advance ➔ **Go to 5**

3 ☐ Something else ➔ **Go to 2**

THE ACCIDENT & EMERGENCY DEPARTMENT

2. When you arrived at the hospital, did you go to the A&E Department (also known as the Emergency Department, Casualty, Medical or Surgical Admissions unit)?

1 ☐ Yes ➔ **Go to 3**

2 ☐ No ➔ **Go to 5**

3. While you were in the A&E Department, how much information about your condition or treatment was given to you?

5 1 ☐ Not enough

10 2 ☐ Right amount

5 3 ☐ Too much

0 4 ☐ I was not given any information about my treatment or condition

- 5 ☐ Don't know / can't remember

4. Were you given enough privacy when being examined or treated in the A&E Department?

10 1 ☐ Yes, definitely

5 2 ☐ Yes, to some extent

0 3 ☐ No

- 4 ☐ Don't know / can't remember

EMERGENCY & URGENTLY ADMITTED PATIENTS, now please go to Question 9

WAITING LIST & PLANNED ADMISSION PATIENTS, please continue to Question 5

WAITING LIST OR PLANNED ADMISSION

5. When you were referred to see a specialist, were you offered a choice of hospital for your **first hospital appointment?**

1 ☐ Yes

2 ☐ No, but I would have liked a choice

3 ☐ No, but I did not mind

4 ☐ Don't know / can't remember

6. How do you feel about the length of time you were on the waiting list before your admission to hospital?

10 1 ☐ I was admitted as soon as I thought was necessary

5 2 ☐ I should have been admitted a bit sooner

0 3 ☐ I should have been admitted a lot sooner

7. Was your admission date changed by the hospital?

10 1 ☐ No

6.7 2 ☐ Yes, once

3.3 3 ☐ Yes, 2 or 3 times

0 4 ☐ Yes, 4 times or more

8. In your opinion, had the specialist you saw in hospital been given all of the necessary information about your condition or illness from the person who referred you?

10 1 ☐ Yes, definitely

5 2 ☐ Yes, to some extent

0 3 ☐ No

- 4 ☐ Don't know / can't remember

ALL TYPES OF ADMISSION

9. From the time you arrived at the hospital, did you feel that you had to wait a long time to get to a bed on a ward?

0 1 ☐ Yes, definitely

5 2 ☐ Yes, to some extent

10 3 ☐ No

THE HOSPITAL & WARD

10. While in hospital, did you ever stay in a critical care area (e.g. Intensive Care Unit, High Dependency Unit or Coronary Care Unit)?

1 ☐ Yes

2 ☐ No

3 ☐ Don't know / can't remember

11. When you were **first** admitted to a bed on a ward, did you share a sleeping area, for example a room or bay, with patients of the opposite sex?

0 1 ☐ Yes

10 2 ☐ No

Q11 and Q13 are scored together to provide a single score on whether patients who have not stayed in a critical care area have ever shared a sleeping area with members of the opposite sex.

Q11 and Q13 are not scored if option 1 ("Yes") is selected to Q10.

Q11 and Q13 score 10 if the respondent did not ever share a sleeping area with patients of the opposite sex, i.e. selected option 2 ("No") to Q11 AND option 2 ("No") to Q13.

If option 1 ("Yes") is selected for EITHER Q11 or Q13 then a score of 0 is assigned.

If ONE of Q11 & Q13 is missing, the other is used for scoring.

12. During your stay in hospital, how many wards did you stay in?

1 ☐ 1 ➔ **Go to 14**

2 ☐ 2 ➔ **Go to 13**

3 ☐ 3 or more ➔ **Go to 13**

4 ☐ Don't know / can't remember ➔ **Go to 14**

13. After you moved to another ward (or wards), did you ever share a sleeping area, for example a room or bay, with patients of the opposite sex?

0 1 ☐ Yes

10 2 ☐ No

14. While staying in hospital, did you ever use the same bathroom or shower area as patients of the opposite sex?

0 1 ☐ Yes

10 2 ☐ Yes, because it had special bathing equipment that I needed

10 3 ☐ No

- 4 ☐ I did not use a bathroom or shower

- 5 ☐ Don't know / can't remember

15. Were you ever bothered by noise **at night** from **other patients**?

0 1 ☐ Yes

10 2 ☐ No

16. Were you ever bothered by noise **at night** from **hospital staff**?

0 1 ☐ Yes

10 2 ☐ No

17. In your opinion, how clean was the hospital room or ward that **you** were in?

10 1 ☐ Very clean

6.7 2 ☐ Fairly clean

3.3 3 ☐ Not very clean

0 4 ☐ Not at all clean

18. How clean were the toilets and bathrooms that **you** used in hospital?

10 1 ☐ Very clean

6.7 2 ☐ Fairly clean

3.3 3 ☐ Not very clean

0 4 ☐ Not at all clean

- 5 ☐ I did not use a toilet or bathroom

19. Did you feel threatened during your stay in hospital by other patients or visitors?

0 1 ☐ Yes

10 2 ☐ No

20. Were hand-wash gels available for patients and visitors to use?

10 1 ☐ Yes

0 2 ☐ Yes, but they were empty

0 3 ☐ I did not see any hand-wash gels

- 4 ☐ Don't know / can't remember

21. How would you rate the hospital food?

10 1 ☐ Very good

6.7 2 ☐ Good

3.3 3 ☐ Fair

0 4 ☐ Poor

- 5 ☐ I did not have any hospital food

22. Were you offered a choice of food?

10 1 ☐ Yes, always

5 2 ☐ Yes, sometimes

0 3 ☐ No

23. Did you get enough help from staff to eat your meals?

10 1 ☐ Yes, always

5 2 ☐ Yes, sometimes

0 3 ☐ No

- 4 ☐ I did not need help to eat meals

DOCTORS

24. When you had important questions to ask a doctor, did you get answers that you could understand?

10 1 ☐ Yes, always

5 2 ☐ Yes, sometimes

0 3 ☐ No

- 4 ☐ I had no need to ask

25. Did you have confidence and trust in the doctors treating you?

10 1 ☐ Yes, always

5 2 ☐ Yes, sometimes

0 3 ☐ No

26. Did doctors talk in front of you as if you weren't there?

0 1 ☐ Yes, often

5 2 ☐ Yes, sometimes

10 3 ☐ No

NURSES

27. When you had important questions to ask a nurse, did you get answers that you could understand?

10 1 ☐ Yes, always

5 2 ☐ Yes, sometimes

0 3 ☐ No

- 4 ☐ I had no need to ask

28. Did you have confidence and trust in the nurses treating you?

10 1 ☐ Yes, always

5 2 ☐ Yes, sometimes

0 3 ☐ No

29. Did nurses talk in front of you as if you weren't there?

0 1 ☐ Yes, often

5 2 ☐ Yes, sometimes

10 3 ☐ No

30. In your opinion, were there enough nurses on duty to care for **you** in hospital?

10 1 ☐ There were always or nearly always enough nurses

5 2 ☐ There were sometimes enough nurses

0 3 ☐ There were rarely or never enough nurses

YOUR CARE & TREATMENT

31. Sometimes in a hospital, a member of staff will say one thing and another will say something quite different. Did this happen to you?

0 ₁ ☐ Yes, often
5 ₂ ☐ Yes, sometimes
10 ₃ ☐ No

32. Were you involved as much as you wanted to be in decisions about your care and treatment?

10 ₁ ☐ Yes, definitely
5 ₂ ☐ Yes, to some extent
0 ₃ ☐ No

33. Did you have confidence in the decisions made about your condition or treatment?

10 ₁ ☐ Yes, always
5 ₂ ☐ Yes, sometimes
0 ₃ ☐ No

34. How much information about your condition or treatment was given to **you**?

0 ₁ ☐ Not enough
10 ₂ ☐ The right amount
0 ₃ ☐ Too much

35. Did you find someone on the hospital staff to talk to about your worries and fears?

10 ₁ ☐ Yes, definitely
5 ₂ ☐ Yes, to some extent
0 ₃ ☐ No
\- ₄ ☐ I had no worries or fears

36. Do you feel you got enough emotional support from hospital staff during your stay?

10 ₁ ☐ Yes, always
5 ₂ ☐ Yes, sometimes
0 ₃ ☐ No
\- ₄ ☐ I did not need any emotional support

37. Were you given enough privacy when discussing your condition or treatment?

10 ₁ ☐ Yes, always
5 ₂ ☐ Yes, sometimes
0 ₃ ☐ No

38. Were you given enough privacy when being examined or treated?

10 ₁ ☐ Yes, always
5 ₂ ☐ Yes, sometimes
0 ₃ ☐ No

39. Were you ever in any pain?

₁ ☐ Yes ➔ **Go to 40**
₂ ☐ No ➔ **Go to 41**

40. Do you think the hospital staff did everything they could to help control your pain?

10 ₁ ☐ Yes, definitely
5 ₂ ☐ Yes, to some extent
0 ₃ ☐ No

41. How many minutes after you used the call button did it usually take before you got the help you needed?

10 ₁ ☐ 0 minutes / right away

7.5 ₂ ☐ 1-2 minutes

5 ₃ ☐ 3-5 minutes

2.5 ₄ ☐ More than 5 minutes

0 ₅ ☐ I never got help when I used the call button

- ₆ ☐ I never used the call button

OPERATIONS & PROCEDURES

42. During your stay in hospital, did you have an operation or procedure?

1 ☐ Yes **➔ Go to 43**

2 ☐ No **➔ Go to 50**

43. Beforehand, did a member of staff explain the risks and benefits of the operation or procedure in a way you could understand?

10 1 ☐ Yes, completely

5 2 ☐ Yes, to some extent

0 3 ☐ No

- 4 ☐ I did not want an explanation

44. Beforehand, did a member of staff explain what would be done during the operation or procedure?

10 1 ☐ Yes, completely

5 2 ☐ Yes, to some extent

0 3 ☐ No

- 4 ☐ I did not want an explanation

45. Beforehand, did a member of staff answer your questions about the operation or procedure in a way you could understand?

10 1 ☐ Yes, completely

5 2 ☐ Yes, to some extent

0 3 ☐ No

- 4 ☐ I did not have any questions

46. Beforehand, were you told how you could expect to feel after you had the operation or procedure?

10 1 ☐ Yes, completely

5 2 ☐ Yes, to some extent

0 3 ☐ No

47. Before the operation or procedure, were you given an anaesthetic or medication to put you to sleep or control your pain?

1 ☐ Yes **➔ Go to 48**

2 ☐ No **➔ Go to 49**

48. Before the operation or procedure, did the anaesthetist or another member of staff explain how he or she would put you to sleep or control your pain in a way you could understand?

10 1 ☐ Yes, completely

5 2 ☐ Yes, to some extent

0 3 ☐ No

49. After the operation or procedure, did a member of staff explain how the operation or procedure had gone in a way you could understand?

10 1 ☐ Yes, completely

5 2 ☐ Yes, to some extent

0 3 ☐ No

LEAVING HOSPITAL

50. Did you feel you were involved in decisions about your discharge from hospital?

10 ₁ ☐ Yes, definitely

5 ₂ ☐ Yes, to some extent

0 ₃ ☐ No

- ₄ ☐ I did not want to be involved

51. Were you given enough notice about when you were going to be discharged?

10 ₁ ☐ Yes, definitely

5 ₂ ☐ Yes, to some extent

0 ₃ ☐ No

52. On the day you left hospital, was your discharge delayed for any reason?

0 ₁ ☐ Yes ➔ **Go to 53**

10 ₂ ☐ No ➔ **Go to 55**

Q52 is used to score Q53 and Q54. See scoring notes under Q53 and Q54 for details.

53. What was the **MAIN** reason for the delay? (**Cross ONE box only**)

0 ₁ ☐ I had to wait for **medicines**

0 ₂ ☐ I had to wait to **see the doctor**

0 ₃ ☐ I had to wait for an **ambulance**

- ₄ ☐ Something else

If response to Q52 is 2 (discharge WAS NOT delayed), Q53 is scored 10.
If response to Q52 is 1 (discharge WAS delayed), and response to Q53 is 1, 2, 3 or 4, the corresponding scores above are assigned to Q53.
If Q52 is missing, Q53 is not scored.
If Q53 is missing, scoring is as per Q52.

54. How long was the delay?

7.5 ₁ ☐ Up to 1 hour

5 ₂ ☐ Longer than 1 hour but no longer than 2 hours

2.5 ₃ ☐ Longer than 2 hours but no longer than 4 hours

0 ₄ ☐ Longer than 4 hours

If response to Q53 is 4 (some other reason for the delay), Q54 is not scored.
If response to Q52 is 2 (discharge WAS NOT delayed), Q54 is scored 10.
If response to Q52 is 1 (discharge WAS delayed) AND the response to Q53 is 1, 2 or 3, the scores above are assigned to Q54.
If response to Q52 is 1 (discharge WAS delayed) AND the response to Q53 is missing, the scores above are assigned to Q54.
If response to Q52 is 1 (discharge WAS delayed) AND the response to Q54 is missing, Q54 is not scored.
If response to Q52 is missing, Q54 is not scored.

55. Before you left hospital, were you given any written or printed information about what you should or should not do after leaving hospital?

10 ₁ ☐ Yes

0 ₂ ☐ No

56. Did a member of staff explain the **purpose** of the medicines you were to take at home in a way you could understand?

10 ₁ ☐ Yes, completely ➔ **Go to 57**

5 ₂ ☐ Yes, to some extent ➔ **Go to 57**

0 ₃ ☐ No ➔ **Go to 57**

- ₄ ☐ I did not need an explanation ➔ **Go to 57**

- ₅ ☐ I had no medicines ➔ **Go to 60**

57. Did a member of staff tell you about medication **side effects** to watch for when you went home?

10 ₁ ☐ Yes, completely
5 ₂ ☐ Yes, to some extent
0 ₃ ☐ No
- ₄ ☐ I did not need an explanation

58. Were you told how to **take** your medication in a way you could understand?

10 ₁ ☐ Yes, definitely
5 ₂ ☐ Yes, to some extent
0 ₃ ☐ No
- ₄ ☐ I did not need to be told how to take my medication

59. Were you given clear written or printed information about your medicines?

10 ₁ ☐ Yes, completely
5 ₂ ☐ Yes, to some extent
0 ₃ ☐ No
- ₄ ☐ I did not need this
- ₅ ☐ Don't know / can't remember

60. Did a member of staff tell you about any danger signals you should watch for after you went home?

10 ₁ ☐ Yes, completely
5 ₂ ☐ Yes, to some extent
0 ₃ ☐ No
- ₄ ☐ It was not necessary

61. Did hospital staff take your family or home situation into account when planning your discharge?

10 ₁ ☐ Yes, completely
5 ₂ ☐ Yes, to some extent
0 ₃ ☐ No
- ₄ ☐ It was not necessary
- ₅ ☐ Don't know / can't remember

62. Did the doctors or nurses give your family or someone close to you all the information they needed to help care for you?

10 ₁ ☐ Yes, definitely
5 ₂ ☐ Yes, to some extent
0 ₃ ☐ No
- ₄ ☐ No family or friends were involved
- ₅ ☐ My family or friends did not want or need information

63. Did hospital staff tell you who to contact if you were worried about your condition or treatment after you left hospital?

10 ₁ ☐ Yes
0 ₂ ☐ No
- ₃ ☐ Don't know / can't remember

64. Did hospital staff discuss with you whether you would need any additional equipment in your home, or any adaptations made to your home, after leaving hospital?

10 ₁ ☐ Yes
0 ₂ ☐ No, but I would have liked them to
- ₃ ☐ No, it was not necessary to discuss it

65. Did hospital staff discuss with you whether you may need any further health or social care services after leaving hospital? (e.g. services from a GP, physiotherapist or community nurse, or assistance from social services or the voluntary sector)

10 ₁ ☐ Yes

0 ₂ ☐ No, but I would have liked them to

\- ₃ ☐ No, it was not necessary to discuss it

OVERALL

66. Overall, did you feel you were treated with respect and dignity while you were in the hospital?

10 ₁ ☐ Yes, always

5 ₂ ☐ Yes, sometimes

0 ₃ ☐ No

67. During your time in hospital did you feel well looked after by hospital staff?

10 ₁ ☐ Yes, always

5 ₂ ☐ Yes, sometimes

0 ₃ ☐ No

68. Overall... (**Please circle a number**)

I had a very poor experience										I had a very good experience
0	1	2	3	4	5	6	7	8	9	10
0	1	2	3	4	5	6	7	8	9	10

69. During your hospital stay, were you ever asked to give your views on the quality of your care?

10 ₁ ☐ Yes

0 ₂ ☐ No

\- ₃ ☐ Don't know / can't remember

70. Did you see, or were you given, any information explaining how to complain to the hospital about the care you received?

10 ₁ ☐ Yes

0 ₂ ☐ No

\- ₃ ☐ Not sure / don't know

ABOUT YOU

71. Who was the main person or people that filled in this questionnaire?

₁ ☐ The **patient** (named on the front of the envelope)

₂ ☐ A **friend or relative** of the patient

₃ ☐ **Both** patient and friend/relative together

₄ ☐ The patient with the help of a health professional

Reminder: All the questions should be answered from the point of view of the person named on the envelope. This includes the following background questions.

72. Are you male or female?

₁ ☐ Male

₂ ☐ Female

73. What was your **year** of birth?

(**Please write in**) e.g. | 1 | 9 | 3 | 4 |

1	9	Y	Y

74. Do you have any of the following long-standing conditions? (**Cross ALL boxes that apply**)

1 ☐ Deafness or severe hearing impairment ➔ **Go to 76**

2 ☐ Blindness or partially sighted ➔ **Go to 76**

3 ☐ A long-standing physical condition ➔ **Go to 76**

4 ☐ A learning disability ➔ **Go to 76**

5 ☐ A mental health condition ➔ **Go to 76**

6 ☐ A long-standing illness, such as cancer, HIV, diabetes, chronic heart disease, or epilepsy ➔ **Go to 76**

7 ☐ No, I do not have a long-standing condition ➔ **Go to 77**

75. Does this condition(s) cause you difficulty with any of the following? (**Cross ALL boxes that apply**)

1 ☐ Everyday activities that people your age can usually do

2 ☐ At work, in education, or training

3 ☐ Access to buildings, streets, or vehicles

4 ☐ Reading or writing

5 ☐ People's attitudes to you because of your condition

6 ☐ Communicating, mixing with others, or socialising

7 ☐ Any other activity

8 ☐ No difficulty with any of these

76. What is your ethnic group? (**Cross ONE box only**)

a. WHITE

1 ☐ English / Welsh / Scottish / Northern Irish / British

2 ☐ Irish

3 ☐ Gypsy or Irish Traveller

4 ☐ Any other White background, **write in......**

[ ]

b. MIXED / MULTIPLE ETHNIC GROUPS

5 ☐ White and Black Caribbean

6 ☐ White and Black African

7 ☐ White and Asian

8 ☐ Any other Mixed / multiple ethnic background, **write in......**

[ ]

c. ASIAN / ASIAN BRITISH

9 ☐ Indian

10 ☐ Pakistani

11 ☐ Bangladeshi

12 ☐ Chinese

13 ☐ Any other Asian background, **write in....**

[ ]

d. BLACK / AFRICAN / CARIBBEAN / BLACK BRITISH

14 ☐ African

15 ☐ Caribbean

16 ☐ Any other Black / African / Caribbean background, **write in.....**

[ ]

e. OTHER ETHNIC GROUP

17 ☐ Arab

18 ☐ Any other ethnic group, **write in......**

[ ]

77. What is your religion?

1 ☐ No religion

2 ☐ Buddhist

3 ☐ Christian (including Church of England, Catholic, Protestant, and other Christian denominations)

4 ☐ Hindu

5 ☐ Jewish

6 ☐ Muslim

7 ☐ Sikh

8 ☐ Other

9 ☐ I would prefer not to say

78. Which of the following best describes how you think of yourself?

1 ☐ Heterosexual / straight

2 ☐ Gay / lesbian

3 ☐ Bisexual

4 ☐ Other

5 ☐ I would prefer not to say

OTHER COMMENTS

If there is anything else you would like to tell us about your experiences in the hospital, please do so here.

Was there anything particularly good about your hospital care?

Was there anything that could be improved?

Any other comments?

Please note that the comments you provide in the box above will be looked at in full by the NHS Trust, CQC and researchers analysing the data. We will remove any information that could identify you before publishing any of your feedback.

THANK YOU VERY MUCH FOR YOUR HELP

Please check that you answered all the questions that apply to you.

Please post this questionnaire back in the FREEPOST envelope provided.
No stamp is needed.
